# PARP-1 negatively regulates nucleolar protein pool and mitochondrial activity: a cell protective mechanism

**DOI:** 10.1186/s41021-024-00312-w

**Published:** 2024-09-18

**Authors:** Atanu Ghorai, Soumajit Saha, Basuthkar J. Rao

**Affiliations:** 1 B-202, Department of Biological Sciences, Tata Institute of Fundamental Research, Homi Bhabha Road, Colaba, Mumbai, 400005 India; 2 Mazumdar Shaw Centre for Translational Research, Mazumdar Shaw Medical Foundation, 8th Floor, ‘A’ Block, 258/A, Bommasandra Industrial Area, Anekal Taluk, Bangalore, 560099 India; 3 Department of Animal Biology, School of Life Sciences, University of Hyderabad, Hyderabad, India

**Keywords:** PARP-1, Nucleolin, Oxidative damage, Nucleolus, Mitochondria, OXPHOS

## Abstract

**Background:**

Poly(ADP-ribose) polymerase-1 (PARP-1) is a pan nuclear protein that utilizes NAD^+^ as a substrate for poly(ADP-ribosyl)ation reaction (PARylation), resulting in both auto-modification and the modification of its accepter proteins. Earlier reports suggested that several nucleolar proteins interact and colocalize with PARP-1, leading to their PARylation. However, whether PARP-1 has any role in nucleolar biogenesis and the functional relevance of such a role is still obscure.

**Results:**

Using PARP-1 depleted cells, we investigated the function of PARP-1 in maintaining the nucleolar morphology and protein levels under normal physiological conditions. Our results revealed that several nucleolar proteins like nucleolin, fibrillarin, and nucleophosmin get up-regulated when PARP-1 is depleted. Additionally, in line with the higher accumulation of nucleolin, stably depleted PARP-1 cells show lower activation of caspase-3, lesser annexin-V staining, and reduced accumulation of AIF in the nucleus upon induction of oxidative stress. Concurrently, PARP-1 silenced cells showed higher mitochondrial oxidative phosphorylation and more fragmented and intermediate mitochondria than the parental counterpart, suggesting higher metabolic activity for better survival.

**Conclusion:**

Based on our findings, we demonstrate that PARP-1 may have a role in regulating nucleolar protein levels and mitochondrial activity, thus maintaining the homeostasis between cell protective and cell death pathways, and such cell-protective mechanism could be implicated as the priming state of a pre-cancerous condition or tumour dormancy.

## Introduction

Nucleolus is a nuclear substructure that is mainly associated with the translational complex assembly. It also has a huge role in ribosomal biosynthesis through the transcription of rDNA genes by RNA polymerase I of a 47 S precursor RNA that is processed further to give rise to 18 S, 5.8 S, and 28 S rRNAs, which then assembles with other ribosomal proteins to form pre-ribosomal particles. Nucleolus is divided into three morphologically distinct zones, reflecting the vectorial process of ribosome biogenesis [1]. The transcription of rDNA occurs at the border of the fibrillar centers (FC) and the dense fibrillar components (DFC), whereas the maturation of the rRNAs and assembly of the pre-ribosomal particles occur in the granular components (GC). Moreover, the nucleolus contains huge varieties of proteins, which are mainly involved in the transcription of rDNA, processing and modification of rRNA transcripts, ribosomal assembly, and transport of the produced ribosome to the cytoplasm [2, 3].

Poly(ADP-ribose) polymerase 1 (PARP-1) is a pan-nuclear enzyme in higher eukaryotes that plays a major role in genome maintenance by participating in DNA damage repair [4–6]. PARP-1 utilizes NAD^+^ as a substrate to generate poly(ADP-ribose) polymers (PAR), ranging from 2 to 200 units, for automodification as well as post-translational modification of acceptor proteins, for example, chromatin-associated histone proteins [7–11]. Poly(ADP-ribosyl)ation (PARylation) of acceptor proteins leads to a change in their localization and function in the cell [12–15]. Hence, to maintain the homeostasis of PARylation and active PARP-1 protein levels, PAR from the poly(ADP-ribosyl)ated proteins are removed and subsequently metabolized by poly(ADP-ribose) glycohydrolase (PARG) [16–19]. Knockout of functional PARG resulted in the accumulation of automodified PARP-1 and modified acceptor proteins, which became incapable of re-associating with DNA and carrying out their normal biological activities, respectively [19, 20].

Though PARP-1 does not contain any known nucleolar localization signal, accumulation of PARP-1 in the nucleolus of interphase cells was earlier reported [21–23]. Proteomic analysis of the nucleolus also revealed the presence of PARP-1 [24, 25]. Different nucleolar proteins from different regions of the nucleolus, such as fibrillarin [11], nucleolin/C23, and nucleoplasmin/B23, were found to interact and colocalize with PARP-1 [26, 27]. Several of them were reported to be poly(ADP-ribosyl)ated also [28]. Additionally, PARP-1 was also known to interact with a number of ribosomal proteins [29, 30]. Both the nucleolar localization and interaction with nucleolar proteins suggest that PARP-1 may have an important function in regulating nucleolar protein levels and biosynthesis.

In the current study, using a set of three human skin fibroblast cell lines system, namely, GMU6 (wild type), GMSiP (PARP-1 stable knockdown), and GMRSiP (Flag-tagged PARP-1 reintroduced in GMSiP background) which are isogenic except for their PARP-1 status, we studied the nucleolar biology. We found out that PARP-1 depletion not only led to translational up-regulation of several nucleolar proteins but also up-regulated nucleolar protein transcription itself. Also, PARP-1 knockdown cells were resistant to cell death compared to wild-type cells upon induction of oxidative stress. It could be possible due to their higher mitochondrial activity which led to showing anti-apoptotic properties, ultimately reinforcing cell-protective measurements.

## Materials and methods

### Cell lines, growth conditions, and treatment

GMU6 (PARP-1 positive cells), GMSiP (PARP-1 stable depleted cells), and GMRSiP (PARP-1 reintroduced in GMSiP background) were a kind gift from Dr. Girish Shah, CHUL, Quebec, Canada [31]. They were diploid human skin fibroblasts GM00637 (Coriell Cell Repository, Camden, NJ), which were cultured in MEM (Gibco) supplemented with 10% FBS (HyClone) and 1× antibiotic-antimycotic (Sigma Aldrich). Cells were grown in a 37 °C incubator with 5% CO_2_ and maintained in the presence of 200 µg/ml hygromycin. To generate ROS inside the cells, which will eventually give rise to oxidative stress, we treated all the cells with 30% H_2_O_2_ diluted in the cell media at a final concentration of 0.5 mM (low dose) and 2 mM (high dose) for 1 h.

### Protein extraction (nuclear and cytoplasmic fractionation), SDS-PAGE, and western blotting

After trypsinization, cells were collected and fractionated, as mentioned in our earlier protocol [32]. Equal amounts of protein (20 µg) were loaded, and the western blot was performed. Primary antibodies like rabbit anti-nucleolin (Abcam), mouse anti-nucleophosmin (Abcam), rabbit anti-fibrillarin (Abcam), rabbit anti-PARP1 (Abcam), rabbit anti-Bcl2XL (Abcam), rabbit anti-Bcl2 (Abcam), mouse anti-AIF (Thermo Fisher Scientific), mouse anti-cleaved caspase-3 (Sigma-Aldrich), and anti-beta actin (Sigma-Aldrich) were used to probe and detect respective proteins.

### Immunoprecipitation

Cells were washed with PBS and subsequently lysed in ice-cold TNN buffer [50 mM Tris-HCl (pH 7.5), 150 mM NaCl, 1% NP 40, 1× protease inhibitor cocktail] for 30 min with periodical mixing. Protein content was determined by BCA assay (Thermo Fisher Scientific). Pre-cleared lysates containing the same amount of protein for all conditions were incubated with anti-nucleolin antibody (Abcam) and anti-nucleophosmin antibody (Abcam) overnight at 4 °C with gentle rocking, and complexes were pulled down with equilibrated protein-G beads. The protein-G beads were washed with TNN buffer thrice for 15 min each. After extensive washing, the complexes were eluted in laemmli buffer by boiling at 94 °C. Equal volumes of the supernatant from each condition were loaded onto each well followed by standard western blotting to determine the interactor proteins.

### RNA extraction and qPCR

Total RNA was extracted from cells using Trizol (Invitrogen, USA). A standard method for cDNA synthesis followed by SYBR green-based qPCR was performed on a BIO-RAD qPCR machine (Bio-Rad Company, USA). Specific primers were selected for nucleolin (forward: 5’- CCAGCCATCCAAAACTCTGT-3’; reverse: 5’-TAACTATCCTTGCCCGAACG-3’) and actin (forward: 5’-TTCCTGGGCATGGAG TC-3’; reverse: 5’-AGGTCTTTGCGGATGTC-3’).

### Immunofluorescence

The standard immunofluorescence (IF) was performed as reported in our previously published protocol [32] with different primary antibodies [rabbit anti-nucleolin (Abcam) and rabbit anti-fibrillarin (Abcam)] and observed under Zeiss confocal microscope (LSM 510 META).

### Annexin V-FITC staining for apoptosis detection

Cells were collected after treatment and washed with ice-cold PBS, followed by staining with FITC-conjugated Annexin-V (BD Biosciences, USA) for 20 min at room temperature in the dark. Cells were counterstained with DAPI to differentiate the nucleus. The stained cells were imaged by a Zeiss epifluorescence microscope.

### Cellular bioenergetics analysis using seahorse XF24 extracellular flux analyzer

We investigated cellular bioenergetics profiles of three cell types- GMU6, GMSiP, and GMRSiP under normal physiological conditions using XF24 Analyzer (Seahorse Biosciences, USA). In brief, 50,000 cells were seeded in each well on the previous day of the seahorse experiment. To monitor real-time changes in mitochondrial oxidative phosphorylation (OXPHOS), sequential injections of oligomycin (1 µM), FCCP (1 µM), and rotenone (1 µM) were performed through the ports of the seahorse cartridge, and the measurement was taken in terms of oxygen consumption rate (OCR in pmol/min). All the measurements were normalized with protein content using Pierce BCA Protein Assay reagent (Thermo Fisher Scientific Inc., USA). We determined several parameters like basal respiration, maximal respiration, spare respiratory capacity, ATP production, proton leak, and non-mitochondrial oxygen consumption.

### Study of mitochondrial morphology

Cells were seeded on 35 mm tissue culture plates with glass bottoms (SPL Life Sciences, Korea) specially designed for confocal live-cell imaging. After overnight growth, cells were stained with 100 nM MitoTracker Green FM (Thermo Fisher Scientific Inc., USA) for 20 min in complete medium, followed by three washes with pre-warm (37 °C) complete medium. Finally, freshly prepared pre-warm complete MEM with 25 mM HEPES was added to the plates, and images were taken using LSM 510 META Zeiss microscope with 63X oil objective within 10 min. A minimum of 30 cells were counted for each cell type.

### Statistical analysis

Data are shown as mean ± standard error of the mean (SEM) or mean ± standard deviation (S.D). Student’s t-test was used to evaluate significant differences between two groups of data. The significance values were denoted as ‘*’ (0.01 < *p* ≤ 0.05), ‘**’ (0.001 < *p* ≤ 0.01), and ‘***’ (*p* ≤ 0.001).

## Results

### PARP-1 depletion leads to an increase in nucleolar count in skin fibroblast cells

**Fig. 1:**
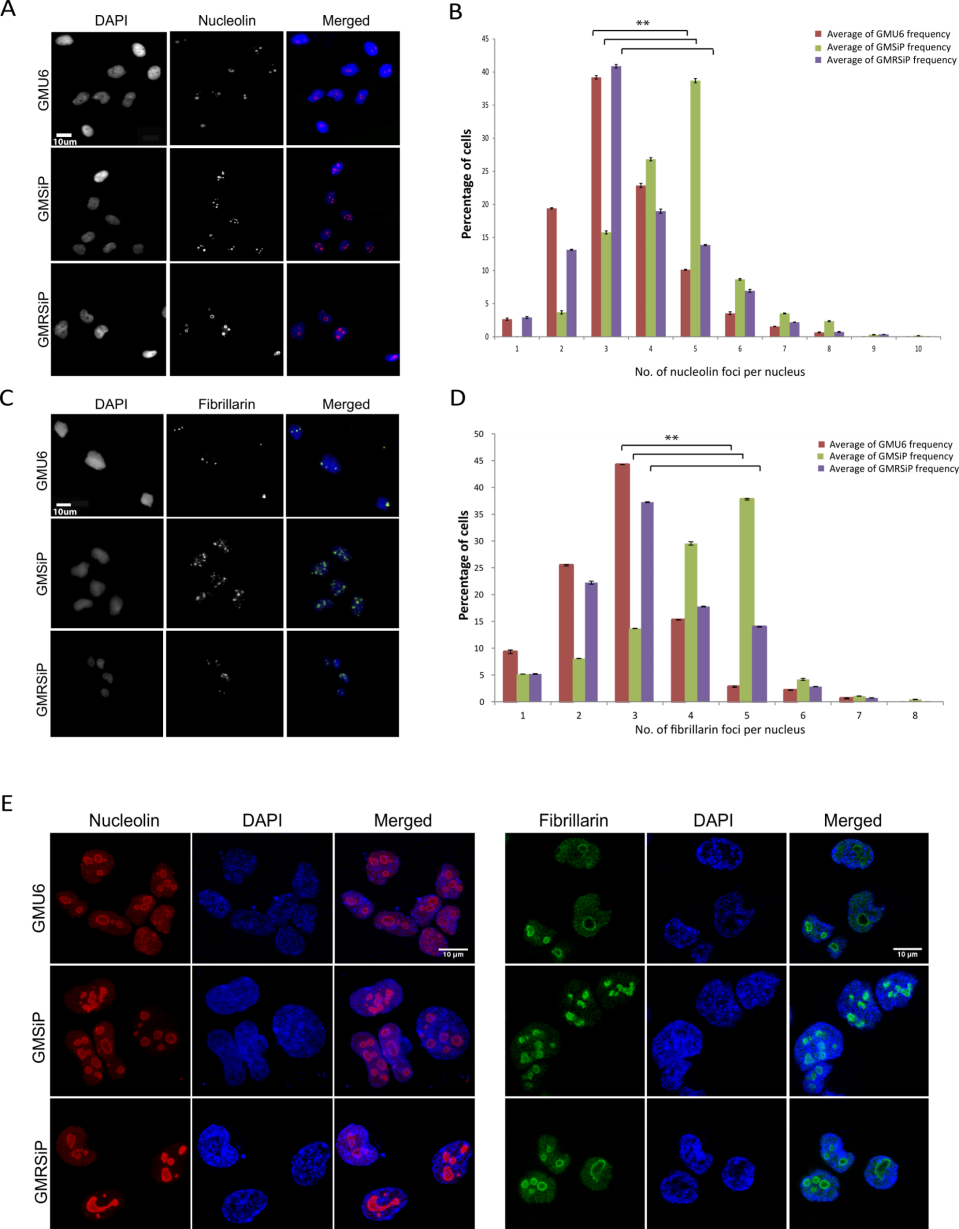
Nucleoli numbers increase in PARP-1 stable knockdown cells. (**A**) The subcellular localization of nucleolin in GMU6, GMSiP, and GMRSiP human fibroblasts as revealed by epifluorescence microscopy (45X magnification). Cells were fixed and stained with rabbit anti-nucleolin monoclonal antibody and imaged. (**B**) Stained nucleolin puncta count was used as an indicator of the nucleoli number per cell. The percentage of cells (Y-axis) with indicated numbers of nucleoli per cell (X-axis) is shown for GMU6 (red bar), GMSiP (green bar), and GMRSiP (purple bar) cells. The data shows mean ± SEM from at least three independent experiments. (**C**) Samples, as in A, were processed and stained with rabbit monoclonal anti-fibrillarin antibody. (**D**) The images were quantified as in B. The data shows mean ± SEM from at least three independent experiments. (**E**) The same experiment was done using a confocal microscope (LSM 510 META Zeiss). The significance values were denoted as ‘**’ (0.001 < *p* ≤ 0.01)

To understand PARP-1 functions in nucleolar biogenesis, we performed immunofluorescence imaging of nucleoli in three fibroblast cell types (GMU6, GMSiP, and GMRSiP) using two nucleolar-specific marker antibodies, namely, nucleolin and fibrillarin. Nucleolin foci number was distinctly higher in PARP-1 knockdown cells (GMSiP) compared to both PARP-1 positive (GMU6 and GMRSiP) cells (Fig. 1A). Quantitation revealed that the majority of GMU6 cells (nearly 40%) exhibited on average about 3 foci/cell. In contrast, GMSiP cells showed a significantly higher count (∼ 5 foci/cell) (Fig. 1B). Fibrillarin staining, which marks the dense fibrillar peripheral portion as opposed to the inner core region of a nucleolus marked by nucleolin protein, corroborated the same result (Fig. 1C & D). Further, we confirmed the same with high-resolution images using confocal microscopy, as shown in Fig. 1E. Thus, it suggests that PARP-1 depletion facilitates higher nucleolar counts in skin fibroblast cells.

### PARP-1 negatively regulates nucleolar protein expression

**Fig. 2:**
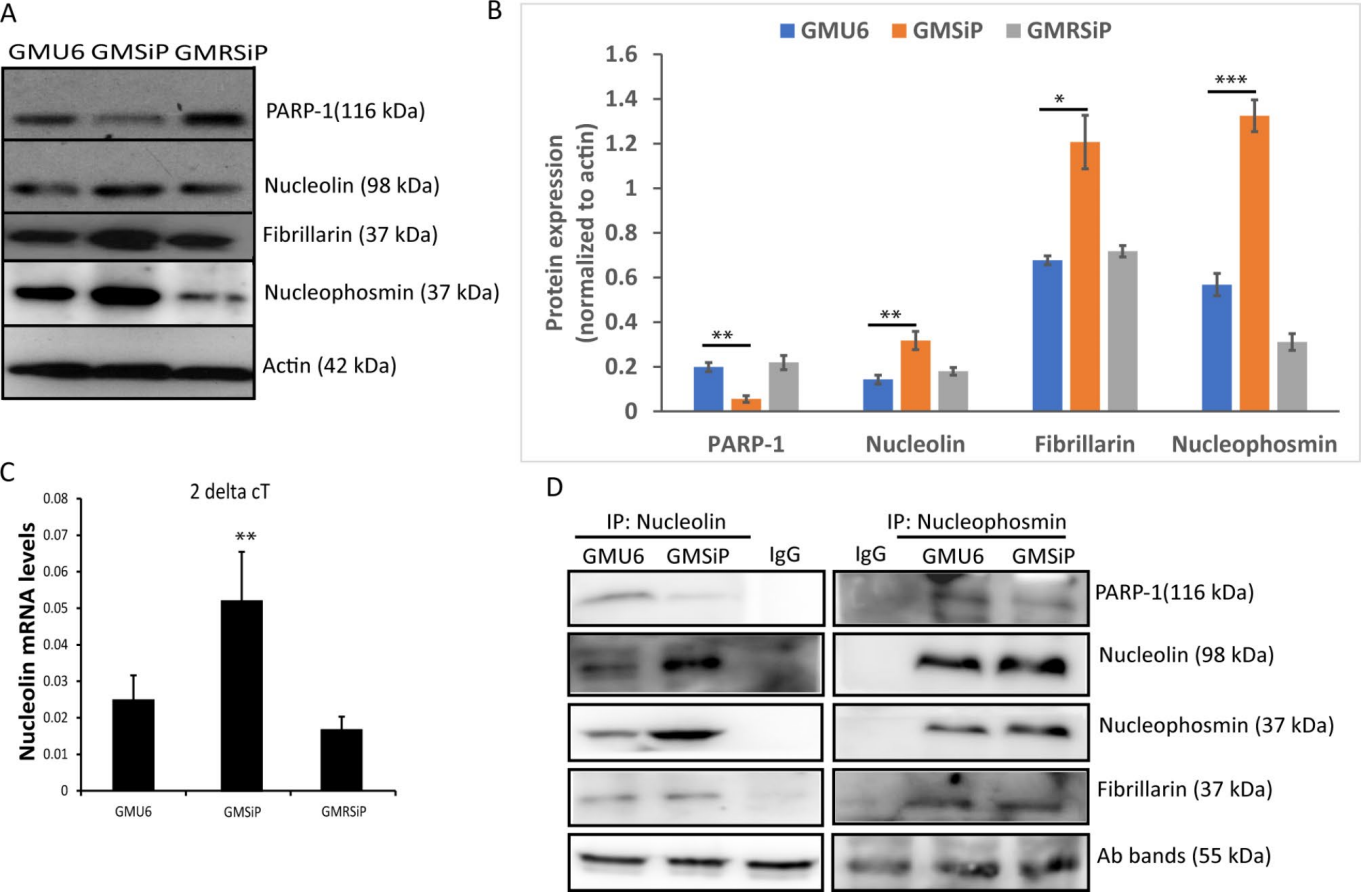
Nucleolar proteins get upregulated in PARP-1 stable knockdown cells and interact with each other. (**A**) Protein lysates from GMU6, GMSiP, and GMRSiP were probed with anti-nucleolin, anti-nucleophosmin, anti-fibrillarin, anti-PARP-1, and anti-actin antibodies. (**B**) The plot showed the respective protein expression in the three types of cells after normalization with actin. (**C**) Total RNA was extracted from GMU6, GMSiP, and GMRSiP cells, and nucleolin mRNA level was measured by qPCR. (**D**) Co-IPed samples using anti-nucleolin and anti-nucleophosmin showed the interaction of PARP-1 and nucleolar proteins with each other. The data shown was the mean ± SD of three independent experiments. The significance values were denoted as ‘*’ (0.01 < *p* ≤ 0.05), ‘**’ (0.001 < *p* ≤ 0.01), and ‘***’ (*p* ≤ 0.001)

To further check whether the increase of nucleolar count per cell in GMSiP is due to the disintegration of nucleolar structures or biosynthesis of new nucleolar structures, we performed immunoblotting in the cell lysates with different nucleolar antibodies. Interestingly, we observed that all the nucleolar markers, e.g. nucleolin, fibrillarin, and nucleophosmin protein levels, were increased in PARP-1 stable depleted cells compared to the control cells (Fig. 2A & B). These results confirmed that the increase in nucleolar punctas in the immunofluorescence study was due to an increase in nucleolar protein levels in GMSiP cells. Lastly, we wanted to confirm whether the increase of nucleolar proteins in GMSiP cells was at the transcriptional or translational level. The qPCR results affirmed that the increase in nucleolar count observed in PARP-1 knockdown cells was accompanied by an about 2-fold rise in the mRNA level of the constituent protein nucleolin compared to that of control PARP-1 positive cells (Fig. 2C), suggesting the possibility of an overall up-regulation of nucleolar mass in PARP-1 depleted cells, an effect counter to that reported in Drosophila studies [33]. Concurrently, we also found that these nucleolar proteins interacted with each other as well as with PARP-1, as shown by the Co-IP study (Fig. 2D). Therefore, our data strongly indicate a PARP-1-mediated negative regulation on nucleolar biogenesis in human skin fibroblasts.

### PARP-1 depleted cells exhibit reduced AIF translocation to nucleus, higher Bcl2/Bcl2-XL level, reduced caspase-3 activation, and protection against cell death after low oxidative stress

**Fig. 3:**
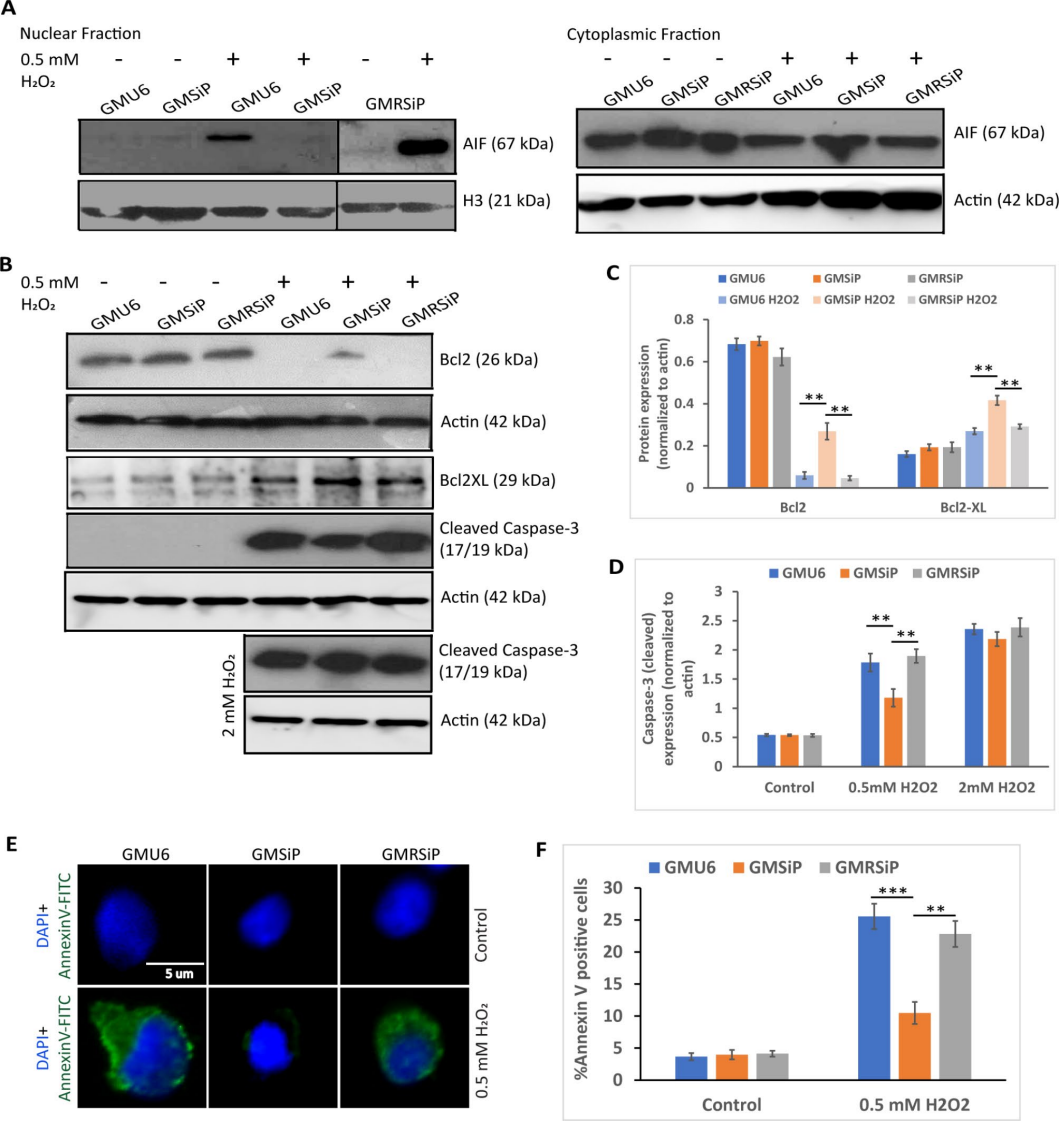
Cell death assays in PARP-1 stable knockdown fibroblast cells compared to PARP-1 positive fibroblasts following oxidative stress. (**A**) The GMU6, GMSiP, and GMRSiP were treated with 0.5 mM of H_2_O_2_, followed by nuclear and cytoplasmic fractionation and immunoblotting using mouse monoclonal anti-AIF antibody to detect AIF translocation. H3 and actin were used as loading controls for nuclear and cytoplasmic fractionation, respectively. (**B**) The three types of cells were treated with 0.5 mM and 2 mM of H_2_O_2_, followed by immunoblotting of the total cell lysates using rabbit anti-Bcl2, rabbit anti-Bcl2XL, and rabbit anti-cleaved caspase-3 antibodies. (**C**) The plot depicted actin normalized expression of Bcl2 and Bcl2XL in GMU6, GMSiP, and GMRSiP after H_2_O_2_ treatment. (**D**) The plot showed actin normalized cleaved caspase-3 expression in three types of cells after H_2_O_2_ treatment with 0.5 and 2 mM. (**E**) Cells treated with 0.5 mM of H_2_O_2_ for 1 h were stained with both Annexin V-FITC and DAPI. Images were acquired in a Zeiss Epifluorescence Microscope with 45X magnification. The scale bar denotes 5 μm. (**F**) The plot showed %Annexin V positive cells after 0.5 mM H_2_O_2_ treatment. The data shown was the mean ± SD of three independent experiments. The significance values were denoted as ‘**’ (0.001 < *p* ≤ 0.01) and ‘***’ (*p* ≤ 0.001)

During parthanatos, a high amount of DNA damage leads to higher activation of PARP-1, which results in the formation of long chains of PAR. The PAR chains are, in turn, removed by PARG (PAR glycohydrolase). The free PAR that is released into the cytosol from the nucleus interacts with a flavoprotein in mitochondria known as AIF (apoptosis-inducing factor). Interaction of AIF with PAR is critical for AIF release from mitochondria into the cytosol [34]. As a result, AIF translocates to the nucleus leading to chromatin condensation, nuclear shrinkage, large-scale DNA fragmentation, and cell death [35–37]. We assessed whether the same holds true in the current cellular paradigm. Following oxidative stress, we observed an efficient translocation of AIF from cytoplasmic to nuclear fraction in PARP-1 positive cells, but the same failed to ensue in PARP-1 knockdown cells (Fig. 3A). This data pointed out that AIF-dependent apoptosis was activated in PARP-1 positive cells following oxidative stress, and as expected, the same was somewhat dampened in PARP-1 knockdown cells. Further, we checked the status of anti-apoptotic mitochondrial proteins Bcl2 and Bcl2-XL in these three cells upon induction of oxidative stress. Immunoblotting revealed that, upon low oxidative stress (0.5 mM H_2_O_2_), both Bcl2 and Bcl2-XL levels were higher in PARP −1 knockdown cells compared to PARP-1 positive cells (Fig. 3B and C). In addition, we also checked the status of cleaved caspase-3 (activated caspase-3) in these cells upon induction of oxidative stress. Caspase-3 activation was less in PARP −1 knockdown cells compared to PARP-1 positive cells after treatment with 0.5 mM H_2_O_2_, as shown in Fig. 3B and D. The cleaved caspase-3 levels became equal in all three cells upon induction with a higher dosage of oxidative stress (2 mM of H_2_O_2_) (Fig. 3B and D).

We further went on to check whether more AIF translocation to the nucleus and more caspase-3 activation upon oxidative stress truly reflect the higher apoptotic nature of PARP-1 positive cells compared to PARP-1 knockdown cells. We co-stained the cells with DAPI and Annexin V-FITC, where the FITC signal will correspond to the amount of cell death (Fig. 3E). As per our expectation, both the PARP-1 positive cells showed a high amount of Annexin-V FITC staining compared to the PARP-1 knockdown cells (Fig. 3F). Together, all the above experiments (high AIF translocation to the nucleus, higher induction of cleaved caspase-3, higher signal of Annexin-V FITC, and lower level of Bcl2 and Bcl2-XL) proved that PARP-1 positive cells indeed are more susceptible to low oxidative stress-mediated cell death compared to PARP-1 knockdown cells, whereas high level of oxidative insult induced comparable level of cell death. Our data corroborates with a recent study where PARP-1 inhibition has been shown to recover cell damage from high oxidative stress-induced cell death [38].

### PARP-1 negatively regulates mitochondrial oxidative phosphorylation (OXPHOS) in human skin fibroblasts

**Fig. 4:**
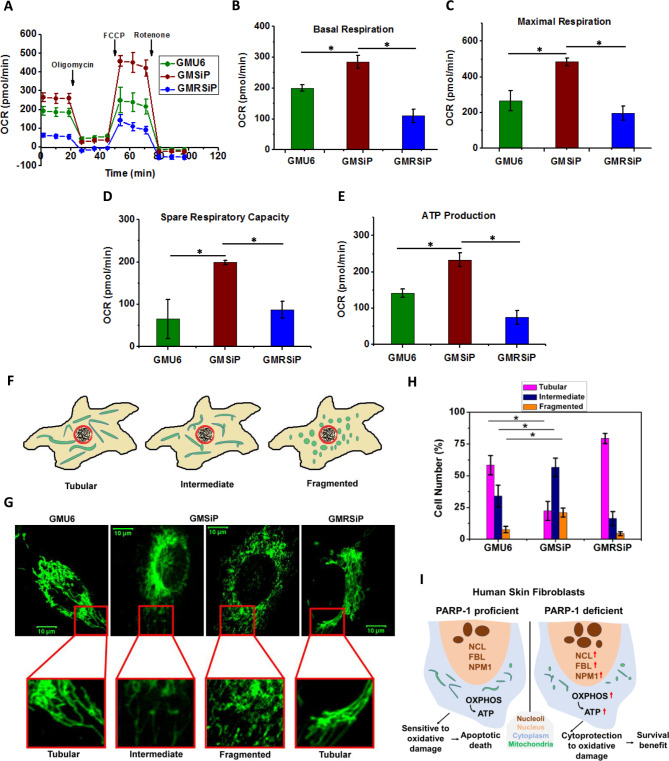
Cellular bioenergetics and mitochondrial morphology dynamics in GMU6, GMSiP, and GMRSiP. Bioenergetics parameters of OXPHOS were determined using extracellular flux analysis (**A-E**). (**A**) OCR measurement was done for 50,000 cells from each well under basal conditions, followed by sequential addition of oligomycin (1 µM), FCCP (1 µM), and rotenone (1 µM). The OXPHOS parameters having significant changes were plotted as basal respiration (**B**), maximal respiration (**C**), spare respiratory capacity (**D**), and ATP production (**E**). The data is shown here as means ± SEM of three independent experiments. Cells were stained with 100 nM MitoTracker Green ^FM^, and live cell imaging was done using LSM 510 META Zeiss microscope (**F-H**). (**F**) Illustration showing the different types of mitochondrial morphology (tubular, intermediate & fragmented) observed and scored in this study. (**G**) Representative images of mitochondria for GMU6, GMSiP, and GMRSiP are shown here. The scale bar is 10 µM. (**H**) Tubular, intermediate, and fragmented mitochondria were counted for each cell type and plotted here as mean ± SD of three independent experiments. The significance values were denoted as ‘*’ (0.01 < *p* ≤ 0.05). (**I**) Illustration depicting the proposed model of the cytoprotective role of PARP-1 during oxidative stress in human skin fibroblasts

The survival benefit of PARP-1 stable knockdown cells against oxidative stress prompted us to check their cellular bioenergetics, which might explain such behaviour of GMSiP cells. We measured and compared various parameters of OXPHOS among these three isogenic cell types. A typical mito stress seahorse profile of GMU6, GMSiP, and GMRSiP is given in Fig. 4A. Intuitively, we found significantly higher basal respiration (Fig. 4B), maximal respiration (Fig. 4C), spare respiratory capacity (Fig. 4D), and ATP production (Fig. 4E) in GMSiP cells compared to parental GMU6 cells. These data suggest that GMSiP cells are more bioenergetically active and handle metabolic stress better than GMU6, which explains their resistance property against low oxidative stress, as shown earlier. Furthermore, our finding corroborates with an existing study where shRNA and siRNA-mediated knockdown of PARP-1 showed higher basal parameters of mitochondrial OXPHOS in cerebral vascular endothelial and lung epithelial cell lines [39]. Interestingly, GMRSiP cells showed lower basal respiration and ATP production than parental GMU6, although maximum respiration and spare respiratory capacity were almost comparable with GMU6, suggesting both GMU6 and GMRSiP behaved similarly under stress which was expected. This result strengthens the role of PARP-1 as a negative regulator of mitochondrial OXPHOS in human skin fibroblast background and might explain the cell protective role of PARP-1 in GMSiP cells at low oxidative stress.

### Effect of PARP-1 depletion on mitochondrial morphology dynamics

We further went ahead to examine whether PARP-1 has any role in mitochondrial morphology dynamics. To do so, we studied mitochondrial morphology by using Mito Tracker Green dye. Mitochondrial morphology is highly dynamic in nature. Under normal physiological conditions, mitochondrial morphology can be tubular, fragmented, and filamentous based on the balancing act between fusion and fission [40]. *Rambold et al.* studied the effect of starvation on mitochondrial morphology by scoring various morphologies like tubulated, fragmented, and intermediate [41]. We also followed the same morphological characterization and scored the mitochondrial morphology among all three cell types in resting conditions as depicted in the cartoon (Fig. 4F). Representative images for different mitochondrial morphologies are given in Fig. 4G. Surprisingly, PARP-1 stable knockdown GMSiP cells showed significantly higher fragmented as well as intermediate mitochondria compared to vector control GMU6 cells as shown in Fig. 4H. We scored the mitochondrial morphology in GMRSiP (PARP-1 is replenished in the GMSiP background), and a significant amount of restoration of mitochondrial phenotype was observed (Fig. 4H). Although we do not have any mechanistic insights behind this, we can assume that PARP-1 might regulate the dynamics of mitochondrial morphology along with the OXPHOS.

## Discussion

The nucleolus is an organelle where ribosomal protein synthesizing machinery accumulates, leading to the processing of rRNA. Due to its primary function, the nucleolus is known to play an important regulator of cell growth in normal and cancer cells [42]. Additionally, the nucleolus also harbors diverse groups of proteins that carry out several biological functions like cell cycle regulation, cell growth, and cell death activation upon induction of DNA damage [43, 44]. Although several previous reports have pointed out that PARP-1 localizes in the nucleolus [26], our study suggests that PARP-1 itself is essential for regulating nucleolar structure and function, particularly for cell death protection against oxidative stress. A plethora of nucleolar factors, including fibrillarin, nucleophosmin, and nucleolin that co-localize in the nucleolus, was seen to localize independently of each other when PARP-1 activity was hindered. Hence, it was suggested that the poly(ADP-ribosyl)ated substrate proteins may serve as a matrix for binding the nucleolar proteins [33]. Our compelling findings further suggest PARP-1 mediated two levels of regulation in nucleolar protein pool dynamics. One is transcriptional level changes (PARP-1 depleted cells, observed in the current study), and another is post-translational level via PAR-mediated scaffolding of nucleolar proteins (PARP-1 positive cells, discussed above).

Here, using a set of three human fibroblast cell line system [31] (isogenic to one another except for their PARP-1 status), we observed that PARP-1 depleted cells exhibited a higher steady-state level of the nucleolar count and enhanced nucleolar protein expression. We were curious about the biological significance of the distinctly higher level of nucleolar proteins observed in normal untreated PARP-1 knockdown cells compared to their PARP-1 positive counterparts and wondered whether PARP-1 protein depletion rather than its presence exerted a general up-regulatory effect on the overall average nucleoli count per se in these cells. At the outset, this effect, if any, seems at odds with the reported findings in the Drosophila system, where it was demonstrated that PARP-1 protein activity was essential for nucleolar biogenesis, their structural maintenance, and pre-ribosome assembly functions in salivary gland tissue [33]. It was also shown in their study that PARP-1 depletion gave rise to nucleolar disintegration, leading to the mislocalization of its constituent proteins to the cytoplasm while maintaining the overall nucleolar protein levels unchanged in the cell. In this context, we reiterate that our experiments with mammalian skin fibroblast cells seem to suggest an opposite effect, where the absence of PARP-1 gave rise to a higher level of nucleolar proteins. At this stage, we point out an important cell biological difference related to PARP-1 protein in Drosophila versus mammalian cells: mammalian PARP-1 protein, unlike that of Drosophila, is much more pan-nuclear in its distribution [26] while in Drosophila, it appears more than 40% of its total nuclear PARP-1 to be specifically at the nucleolar compartment in all types of tissues (salivary gland, diploid brain cells) [33] per se which might hint at the possibility that mammalian PARP-1 protein has acquired additional chromatin-centric roles (non-enzymatic roles like transcription regulation), perhaps less observed in Drosophila, forming the basis of dissimilarity in PARP-1 functions, if any, between these two systems. One report showed PARP-1 mediated positive regulation of ribosomal biogenesis in breast cancer cells, but nucleolar proteins or nucleolar architectures were not discussed [45]. Thus, our findings in normal skin fibroblast cells open up an interesting insight into the involvement of PARP-1 in nucleolar biology.

Among the multitude of protective responses cells put out, those mediated by nucleolar proteins and the associated unfolded protein response via the Hsp70 pathway rank paramount [46]. It has been well documented that nucleolin (C23), one of the major nucleolar anchor proteins, interacts with PARP-1 [28, 47], and plays a critical role in cell protection as an anti-apoptotic component [48] besides functioning in rDNA transcription [49] and cell cycle control [50]. Nucleolin has also been shown to stabilize Bcl-2 during ROS-mediated cellular damage [51], thereby protecting cells from undergoing apoptosis. Our data also revealed that PARP-1 depleted cells showed higher resistance to death as compared to PARP-1 positive cells in oxidative stress by escaping cell death via AIF as well as Bcl2, Bcl2-XL, and caspase-3 dependent mechanisms.

To further understand the physiological and cellular events cumulatively helping PARP-1 depleted cells exhibiting better cell survival, we checked the cellular bioenergetics status of these cells. Mitochondrial energy status is essential for cell survival, even in nuclear genotoxic stress [52]. Hence, our result, together with the previous study [39], indicated that PARP-1 negatively regulates mitochondrial OXPHOS. Higher maximum respiratory and spare respiratory capacity in PARP-1 depleted cells give more advantages to protecting cells against oxidative stress. The cytoprotective role of PARP-1 inhibition was shown earlier in pathophysiological conditions like myocardial ischemia/reperfusion injury/cardiomyocyte injury [53–55]. During such pathological conditions, oxidative stress hyper-activated PARP-1, leading to depletion of NAD^+^ followed by higher necrotic cell death. Here, we showed that cell protection by PARP-1 depletion was achieved somehow by enhancing mitochondrial respiratory functions. Again, mitochondrial energy status may be maintained by the dynamic nature of its morphology, and that is implicated in several disease contexts as well [56]. We found an unanticipated finding of having more fragmented mitochondria in PARP-1 depleted cells (although having higher OXPHOS) compared to wild-type counterparts, suggesting a possible indication of increased mitochondrial fission leading to mitophagy which needs to be tested. Intriguingly, several studies also reported similar paradoxical findings. *Parkin*-mutant fibroblasts derived from Parkinson’s patients showed a higher respiratory rate with a higher fragmented mitochondrial network than normal fibroblasts [57]. Another study reported that cardiac fragmented mitochondria due to increased mitochondrial respiration without mitophagy were induced during regular exercise [58]. Recently, *Jankó et al*. showed that PARP-2 silenced myoblasts had fragmented mitochondria with higher basal oxygen consumption rates and ruled out the involvement of mitophagy [59]. Interestingly, they also mentioned a mild increase of fragmented mitochondria in PARP-1 silenced cells, but mitochondrial OXPHOS was not tested. Therefore, all this evidence further strengthens the cytoprotective role of PARP-1 depletion in skin fibroblasts via mitochondrial connections.

Further, to connect the intricacies of our findings to a broader biological perspective, we presume the characteristics of PARP-1 depleted cells as the ‘pre-neoplastic’ condition where cells are prime to a tumorigenic phenotype. It is well known that cancer progression or tumorigenesis is positively associated with nucleolar biogenesis as well as higher mitochondrial activity to boost the hyper-proliferation in the cancerous system [60–62]. In this regard, our study reveals the strong association of depletion of PARP-1 with cancerous phenotypes (increased level of nucleolar biogenesis, mitochondrial activity or energy, and resistance to apoptosis). It opens up a novel direction of understanding of PARP-1 in carcinogenesis, which has not been reported earlier. However, the knowledge from PARP-1 knockout mice indicates its tumorigenic potential [63, 64] and increased susceptibility to nitrosamine carcinogenicity [65]. Notably, it implies that PARP-1 plays a balancing act between switching a normal cell into a pre-cancerous stage. Therefore, the cell-protective role of PARP-1 (against mild oxidative stress, as evident from this study) could have been implicated in those therapy-resistant cancer cells where cells survive in a dormancy stage (although they are metabolically active) but grow back with tumorigenic potential [66]. Further, cancer cells prefer glycolytic over OXPHOS for their survival, known as the Warburg effect. However, recent studies also showed OXPHOS-dependent metabolic rewiring in therapeutic drug-treated cancer cells, leading to the evolution of a resistant population [67–70]. PARP-1 inhibitors, such as Olaparib-resistant tumours have been reported, which is a serious issue in cancer therapy [71]. From our study, we can presume that PARP-1 inhibitor resistant tumours could have higher OXPHOS and nucleolar protein pools, which would be exciting targets for the combinatorial strategy to deal with such therapy-resistant tumours.

Overall, our findings together indicate that PARP-1 is critical for nucleolar protein level maintenance (both transcriptionally and translationally) and cellular protection against oxidative stress by regulating mitochondrial activity, which could have been attributed to the pre-cancerous potential of cells.

## Conclusion

In summary, we have shown that PARP-1 negatively regulates nucleolar protein expression and mitochondrial activity. Taken together, our study enhances the understanding of PARP-1 involvement in normal cellular and physiological conditions apart from its well-established DNA repair role. A more in-depth investigation into the interconnection of ribosomal biogenesis and mitochondrial activity via PARP-1 would significantly contribute to uncovering novel therapeutic approaches and the development of targeted therapies to deal with PARP-1-associated pathophysiological conditions in the future.

## Data Availability

There are no large datasets generated in this study. All biological materials will be made available upon reasonable request from the corresponding author Basuthkar J Rao (bjrao@uohyd.ac.in).
